# MXene/Carboxymethyl Chitosan Moisture Responsive Soft Actuator with Diode‐Like Actuation for Versatile Applications Driven by Human Metabolism

**DOI:** 10.1002/advs.202507845

**Published:** 2025-07-21

**Authors:** Liangliang Xu, Yangyang Ling, Ziqing Li, Xinyu Xu, Xiaoxia Li, Longfei Chang, Qingyu Peng, Ying Hu

**Affiliations:** ^1^ Anhui Province Key Lab of Aerospace Structural Parts Forming Technology and Equipment School of Materials Science and Engineering Hefei University of Technology Hefei 230009 P. R. China; ^2^ National Key Laboratory of Science and Technology on Advanced Composites in Special Environments Center for Composite Materials and Structures Harbin Institute of Technology Harbin 150080 P. R. China

**Keywords:** asymmetric structure, diode deformation, moisture responsive, soft actuator, soft robotics

## Abstract

Moisture responsive soft actuators are receiving increasing attention due to their unique potential in reducing external energy dependence and carbon footprint. For the conventional moisture responsive soft actuators, their bending deformation under moisture stimulation is usually bidirectional, and the orientation of the bending axis is random. Achieving a moisture responsive monolithic actuator with controllable unidirectional deformation remains a challenge. Here, a Ti_3_C_2_T_x_ MXene/carboxymethyl chitosan composite film actuator with thickness gradient along length direction is fabricated via a vacuum‐assisted “tilt‐filtration” approach. The actuator exhibits a “diode‐like” controllable unidirectional deformation behavior under moisture gradient, and its deformation direction is strictly correlated to its thickness gradient direction and moisture source direction. Based on this highly correlated actuation behavior with internal structural asymmetry, a self‐sustained oscillator under a constant moisture gradient is achieved. Besides, various multifunctional applications based on this actuator driven by human metabolism are also demonstrated, including non‐contact switch with unidirectional conductivity, intelligent keyboard for non‐contact character input, biomimetic crawling robot, wearable intelligent thermal management clothing, and a self‐powered respiratory sensor. This work paves the way for the realization of moisture responsive soft actuators with unidirectional controllable deformation, and further promotes the development of sustainable intelligent materials in soft robotics and electronics.

## Graphical abstract

A MXene‐based actuator with gradient thickness is fabricated by a novel and simple vacuum‐assisted “tilt‐filtration” approach. The actuator exhibits a “diode‐like” controllable unidirectional deformation behavior under moisture gradient, and its deformation direction is strictly correlated to its thickness gradient direction and moisture source direction. Based on this actuator, a series of versatile applications driven by human metabolism are demonstrated.

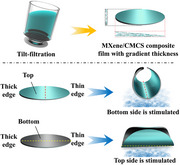

## 1 Introduction

Soft actuators that can convert the energy generated by external stimuli (e.g., heat,^[^ ^1^, ^2^ ^]^ light,^[^ ^3^, ^4^ ^]^ electric,^[^ ^5^, ^6^ ^]^ humidity,^[^ ^7^, ^8^, ^9^, ^10^ ^]^ magnetic,^[^ ^11^, ^12^ ^]^ chemical vapor,^[^ ^13^, ^14^ ^]^ etc.) into mechanical deformation have shown great potential for applications in fields such as artificial muscles,^[^ ^9^, ^15^, ^16^ ^]^ soft robotics,^[^ ^1^, ^3^, ^11^, ^17^, ^18^ ^]^ and intelligent electrical devices.^[^ ^19^, ^20^, ^21^ ^]^ With the increasing global demand for green energy and sustainable technologies, the development of low‐energy and environmentally friendly soft actuators has become crucial. Compared with other forms of stimulation, moisture responsive soft actuators have shown unique potential in reducing external energy dependence and carbon footprint by directly utilizing the widely existing moisture in nature as an energy source.^[^ ^22^, ^23^ ^]^ In addition to being widely present in nature, humans can also excrete a large amount of water every day through skin evaporation or exhalation. Using the water excreted by the human body's own metabolism as the driving source for soft actuators, without the need for external energy input or the production of harmful substances, can achieve the output of mechanical energy, which means that it can serve as a truly green and non‐consumable future power source.

The actuation mechanism of the moisture responsive actuators is that when the environmental humidity changes, the actuator absorbs/desorbs water molecules, resulting in an asymmetric change in volume, which leads to the actuation behavior of the actuator.^[^ ^22^, ^24^ ^]^ Taking the thin‐film‐type moisture responsive actuator as an example, when one side of the film is stimulated by moisture, the film will bend toward the other side with lower humidity.^[^ ^25^, ^26^ ^]^ Due to the uncontrolled microstructure, the orientation of bending axis of the film actuator is random. In order to achieve high‐performance actuation behavior, it is usually necessary to apply moisture stimulation to the obtained sample to confirm the bending axis orientation, and then cut it for further use. This process is tedious. In addition, the deformation of the current moisture responsive actuators is usually bidirectional. When moisture stimulation is applied to the other side of the film, the film will undergo bending deformation in the opposite direction. However, in certain application scenarios, we expect the actuators to only undergo deformation in a specific direction when stimulated. For example, soft robots with unidirectional deformation can be preset with motion directions,^[^ ^27^ ^]^ and unidirectional intelligent microfluidic valves based on soft actuators can control the direction of liquid flow to avoid backflow contamination.^[^ ^28^ ^]^ In order to achieve controllable unidirectional deformation of the thin‐film‐type moisture responsive actuator, current measures include introducing additional moisture insensitive limiting layers (such as polymer layers) to obtain bilayer or multilayer structures, or patterned processing to obtain asymmetric structures. However, bilayer or multilayer heterostructures face problems such as complex manufacturing processes, unstable interfaces, and poor reliability. Pattern processing has high requirements for design schemes and equipment accuracy. Achieving a moisture gradient responsive monolithic actuator with controllable unidirectional deformation in a simple, universal, and low‐cost manner remains a challenge.

In this work, a moisture responsive Ti_3_C_2_T_x_ MXene/carboxymethyl chitosan (MXene/CMCS) composite film actuator with gradient thickness along length direction is fabricated by a simple and novel vacuum‐assisted “tilt‐filtration” method. The terminal polar functional groups of Ti_3_C_2_T_x_ MXene make it excellent hydrophilicity and can easily adsorb water molecules through hydrogen bonding.^[^ ^29^, ^30^, ^31^ ^]^ It can increase the interlayer spacing of nanosheets without changing its crystal structure, and has been proven to be one of the ideal materials for achieving moisture responsive soft actuators.^[^ ^8^, ^30^, ^31^, ^32^, ^33^ ^]^ Carboxymethyl chitosan, as a biodegradable natural polymer derivative, has a large number of hydrophilic groups that enable it to strongly bind with MXene,^[^ ^34^, ^35^ ^]^ enhancing its mechanical properties and water absorption and swelling ability. Based on these, this MXene/CMCS composite film is highly sensitive to moisture changes and can generate rapid driving deformation under moisture gradient. It is very important that, unlike the random orientation bending deformation of the conventional moisture responsive composite film actuators, this MXene/CMCS composite film actuator with gradient thickness exhibits a “diode‐like” controllable unidirectional deformation behavior under moisture gradient, and its deformation direction is strictly correlated with the directions of the thickness gradient and the moisture source. When the moisture comes from the bottom side of the composite film, the composite film undergoes bending deformation toward the top side, and its bending axis is the symmetry axis perpendicular to the thickness gradient direction of the composite film. When the moisture source comes from the top side of the composite film, the composite film deforms toward the bottom side, and its bending axis becomes the symmetrical axis parallel to the thickness gradient direction. Based on this highly correlated actuation behavior with internal structural asymmetry of this composite film, a self‐sustained oscillation device under a constant moisture gradient is achieved. In addition, various multifunctional applications based on this MXene/CMCS composite film using moisture from human skin evaporation or exhalation as energy source are demonstrated, including non‐contact switches with unidirectional conductivity and light/moisture dual response, intelligent keyboard for non‐contact character input, soft biomimetic crawling robot, wearable intelligent thermal management clothing, and self‐powered sensor that can recognize respiratory status. This work provides a new strategy for achieving moisture responsive soft actuators with controllable unidirectional deformation, and its multifunctional applications based on human metabolism as the driving source further promotes the development of sustainable materials in green, low‐energy soft intelligent devices and robotics.

## 2 Results and Discussion

### 2.1 Fabrication and Characterization of the MXene/CMCS Composite Film with Gradient Thickness

**Figure 1:**
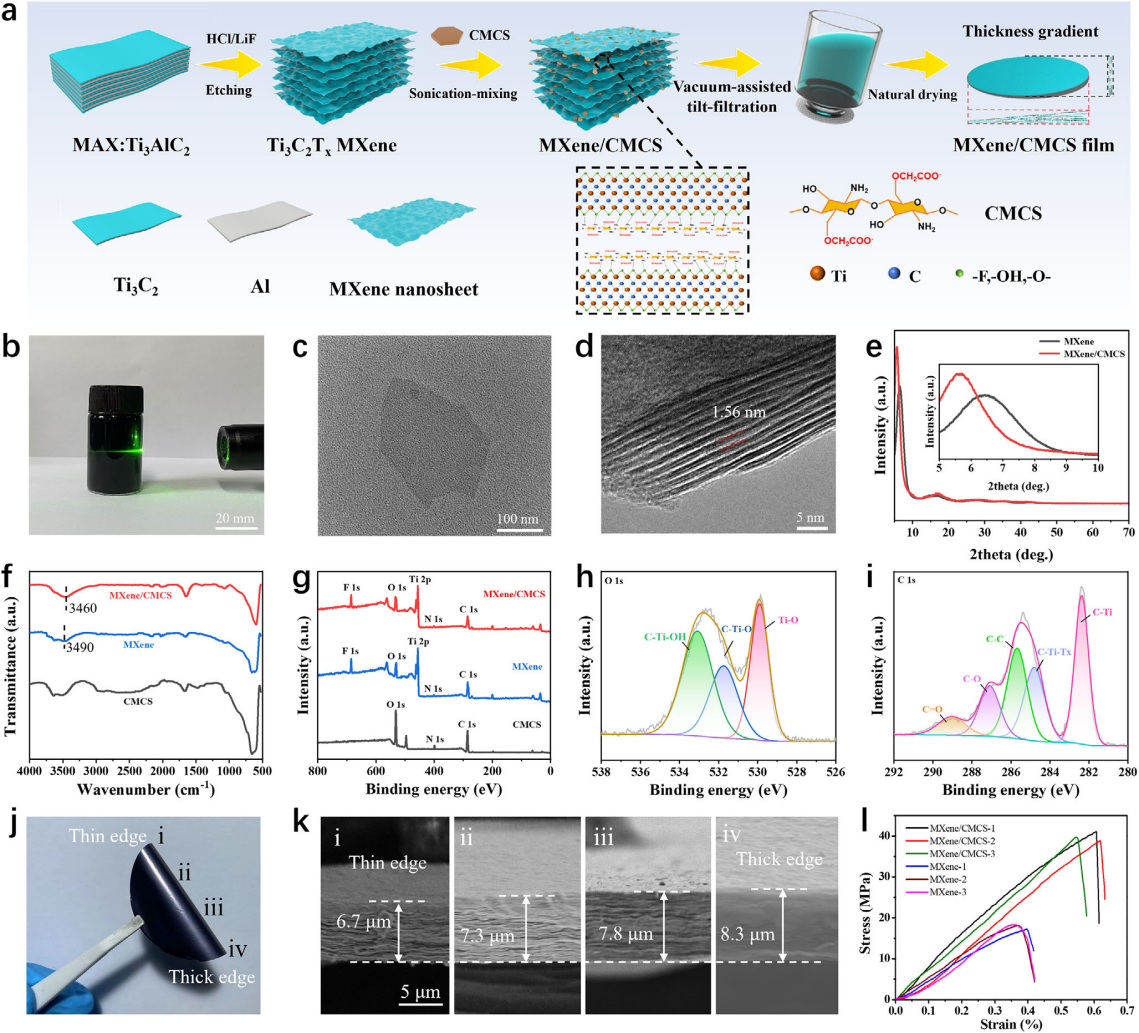
Fabrication and characterization of the MXene/CMCS composite film with gradient thickness. a) Schematic illustration of the fabrication procedure of the MXene/CMCS composite film with gradient thickness. b) Tyndall effect of the Ti_3_C_2_T_x_ MXene dispersion. c) TEM image of the Ti_3_C_2_T_x_ MXene nanosheet. d) HRTEM image of the MXene/CMCS composite material. e) XRD patterns of Ti_3_C_2_T_x_ MXene and MXene/CMCS. f) FTIR spectra and g) XPS spectra of Ti_3_C_2_T_x_ MXene, CMCS, and MXene/CMCS. h) High‐resolution XPS spectra of MXene/CMCS in O1s region and i) C1s region. j) Optical image of the MXene/CMCS composite film. k) Cross‐sectional SEM images of the MXene/CMCS composite film at different positions. l) Stress–strain curves of MXene films and MXene/CMCS composite films.

A vacuum‐assisted “tilt‐filtration” approach is used to fabricate the MXene/CMCS composite film with gradient thickness. Unlike the conventional vacuum‐assisted filtration method, in this work, the filtration device is slightly tilted (with a tilt angle of ≈12°). During the “tilt‐filtration” process, MXene/CMCS composite tend to aggregate toward the lower regions, resulting in a thickness gradient in the obtained composite film. **Figure** **1**a schematically shows the fabrication process of the moisture responsive MXene/CMCS composite film with gradient thickness. Ti_3_C_2_T_x_ MXene is synthesized by a typical HCl/LiF chemical etching method (the detailed synthesis process is given in the Experimental Section). Tyndall effect of the MXene dispersion under laser irradiation can be observed clearly (Figure 1b), which indicates the good water dispersibility of the Ti_3_C_2_T_x_ MXene nanosheets. The transmission electron microscopy (TEM) image shows the typical two‐dimensional sheet structure of the synthesized Ti_3_C_2_T_x_ MXene (Figure 1c). The Ti_3_C_2_T_x_ MXene dispersion is slowly added into a CMCS aqueous solution and magnetic stirred continuously to obtain the MXene/CMCS mixed dispersion. Subsequently, the mixed dispersion is subjected to vacuum‐assisted “tilt‐filtration”, and the MXene/CMCS composite film with gradient thickness can be obtained after natural drying.

The high‐resolution TEM (HRTEM) images of MXene and MXene/CMCS composite material indicate that the introduction of CMCS increases the interlayer spacing of Ti_3_C_2_T_x_ MXene from 1.39 to 1.56 nm (Figure 1d; Figure S1, Supporting Information). X‐ray diffraction (XRD) pattern further confirms the increasing of the interlayer spacing of the MXene nanosheets. As shown in Figure 1e, the characteristic peak corresponding to the (002) lattice plane of the MXene nanosheets is decreased from 6.4 to 5.7°. Fourier transform infrared (FTIR) spectroscopy is used to confirm the successful assembly of CMCS onto MXene nanosheets (Figure 1f). Compared with the pure Ti_3_C_2_T_x_ MXene, the stretching vibration peak of ‐OH of the MXene/CMCS composite material shifts from 3490 to 3460 cm^−1^, indicating a strong hydrogen bonding interaction between CMCS and MXene nanosheets.^[^ ^34^ ^]^ X‐ray photoelectron spectroscopy (XPS) is also measured to analyze the chemical bonding between Ti_3_C_2_T_x_ MXene and CMCS (Figure 1g). In the high‐resolution O 1s spectrum of MXene/CMCS composite material, C‐Ti‐OH (533.08 eV), C‐Ti‐O (531.74 eV), and Ti‐O (529.90 eV) are identified respectively (Figure 1h). As a comparison, the high‐resolution XPS spectrums of pure Ti_3_C_2_T_x_ MXene are also given (Figure S2, Supporting Information). The introduction of CMCS effectively enhances the C─Ti─OH peak, which can be attributed to the abundant hydroxyl groups in CMCS enabling strong binding with MXene nanosheets. The peaks at 289.04, 287.10, 285.68, 284.80, and 282.37 eV in the high‐resolution C 1s spectrum of the MXene/CMCS composite material correspond to C═O, C─O, C─C, C─Ti Tx, and C─Ti, respectively (Figure 1i). The introduction of CMCS also enhances the C═O and C─O peaks. The high‐resolution Ti 2p and F 1s spectrums of MXene/CMCS are also provided in the supporting information (Figure S3, Supporting Information). Figure 1j gives the optical image of the MXene/CMCS composite film, which demonstrates its excellent mechanical flexibility. The cross‐sectional scanning electron microscopy (SEM) images of the MXene/CMCS composite film at different positions are given in Figure 1k, displaying clear layered structures and distinct thickness gradient. The thickness of the thick edge and thin edge of MXene/CMCS composite film is ≈8.3 and ≈6.7 µm, respectively. Figure 1l gives the stress‐strain curves of the MXene/CMCS composite films and MXene films (3 samples for each material), and the results indicate that the introduction of CMCS can significantly increase the tensile strength of MXene film (from 17.9 ± 0.5 to 39.9 ± 0.9 MPa).

### 2.2 “Diode‐Like” Actuation Behavior of the MXene/CMCS Composite Film

**Figure 2:**
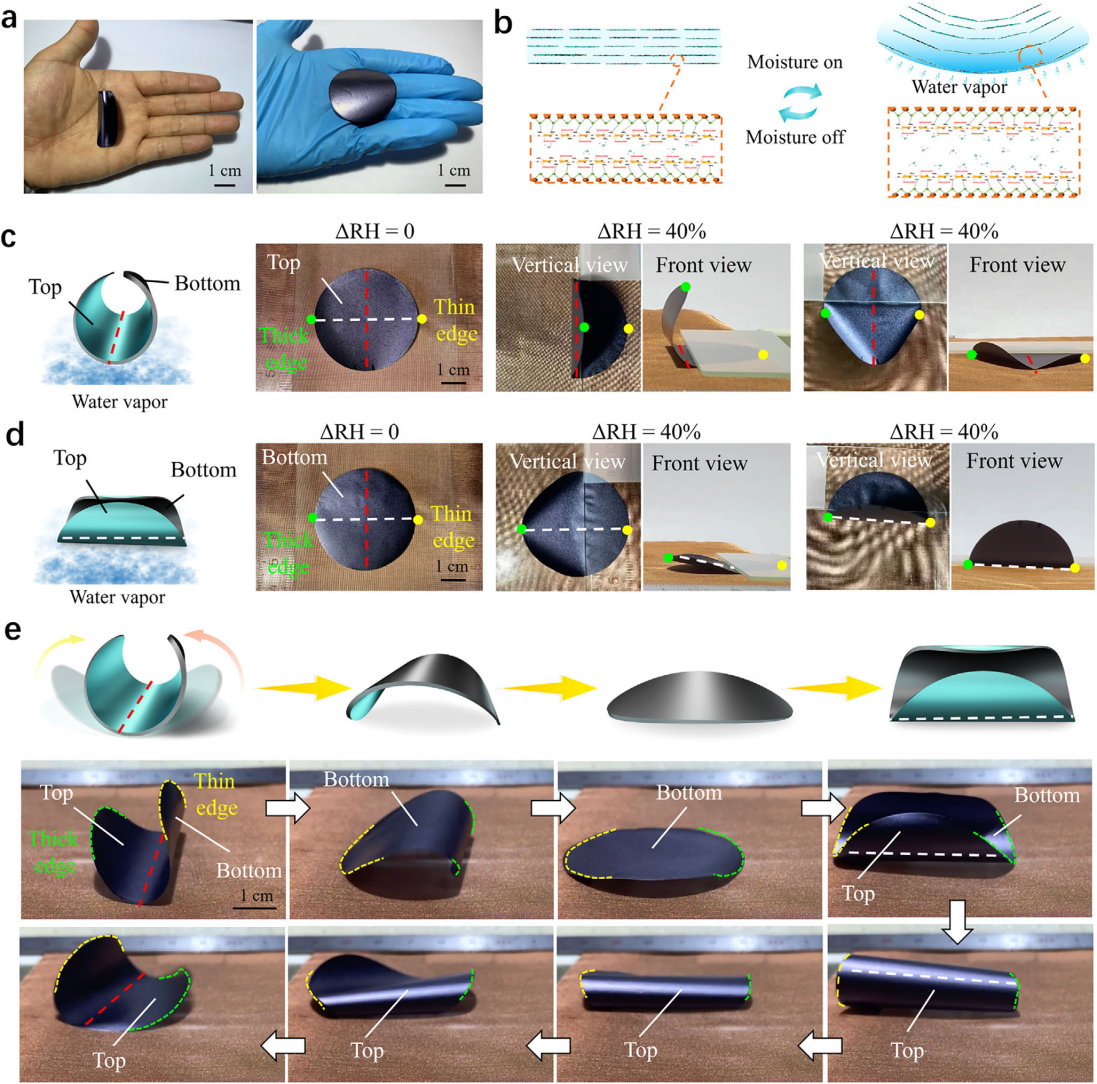
Actuation performance of the MXene/CMCS composite film with gradient thickness. a) Deformation behavior of composite film on human palm. b) Actuation mechanism of the composite film under moisture gradient. c) Deformation behavior of the composite film when its bottom side is stimulated by moisture. d) Deformation behavior of the composite film when its top side is stimulated by moisture. e) Periodic flipping motion of the composite film under continuous moisture supply. The bending axis of the composite film is highly correlated to its thickness gradient direction and moisture source direction.

Like most MXene‐based film actuators, this MXene/CMCS composite film exhibits moisture responsive driving deformation capability. As shown in **Figure** **2**a, when the composite film is placed on a human palm, it immediately undergoes significant bending deformation toward the air side. When it is placed on a gloved palm, it does not achieve significant deformation. The actuation behavior of the MXene/CMCS composite film is attributed to the asymmetric expansion on both sides of the composite film caused by the moisture gradient. Due to the water molecules sensitive interlayer spacing variation of Ti_3_C_2_T_x_ MXene and the water absorption ability caused by the abundant hydroxyl functional groups of CMCS, the side of the MXene/CMCS composite film near the palm will absorb water molecules and undergo expansion deformation. Water molecules cannot pass through the composite film within a limited time, so the side of the composite film near the air will not experience significant expansion. The asymmetric deformation on both sides leads to bending deformation of the composite film toward the air side (Figure 2b).

It is worth noting that, unlike the irregular deformation of the moisture responsive film actuators obtained by conventional vacuum‐assisted filtration method, this MXene/CMCS composite film fabricated by “tilt‐filtration” approach exhibits a “diode‐like” actuation behavior, which is highly correlated with the direction of its thickness gradient and the direction of the moisture source. The side of the composite film obtained in contact with the filter membrane is named the bottom side, and the side in contact with the air is named the top side. The MXene/CMCS composite film is placed on a copper mesh with continuous moisture supply below, and a glass sheet is pressed onto a part of the composite film. As shown in Figure 2c, when the bottom side of the composite film contacts with the copper mesh, the moisture supply from below the copper mesh causes the composite film to undergo upward bending deformation, with the bending axis being the symmetry axis of the composite film perpendicular to its thickness gradient direction. It can be seen that no matter how the position of the glass sheet is changed, the free part of the composite film always undergoes bending deformation toward the axis of symmetry perpendicular to the thickness gradient direction of the composite film. When the top side of the composite film contacts with the copper mesh, the free part of the composite film still undergoes upward bending deformation, but its bending axis changes to the symmetry axis parallel to the thickness gradient direction (Figure 2d). After removing the glass sheet, the composite film can achieve periodic flipping motion under continuous moisture supply (Figure 2e; Movie S1, Supporting Information), and the bending axis of each flip is consistent with the aforementioned phenomenon. That is, when the bottom side contacts the copper mesh, it flips along the axis of symmetry perpendicular to its thickness gradient direction, and when the top side contacts the copper mesh, it flips along the axis of symmetry parallel to the thickness gradient direction.

**Figure 3:**
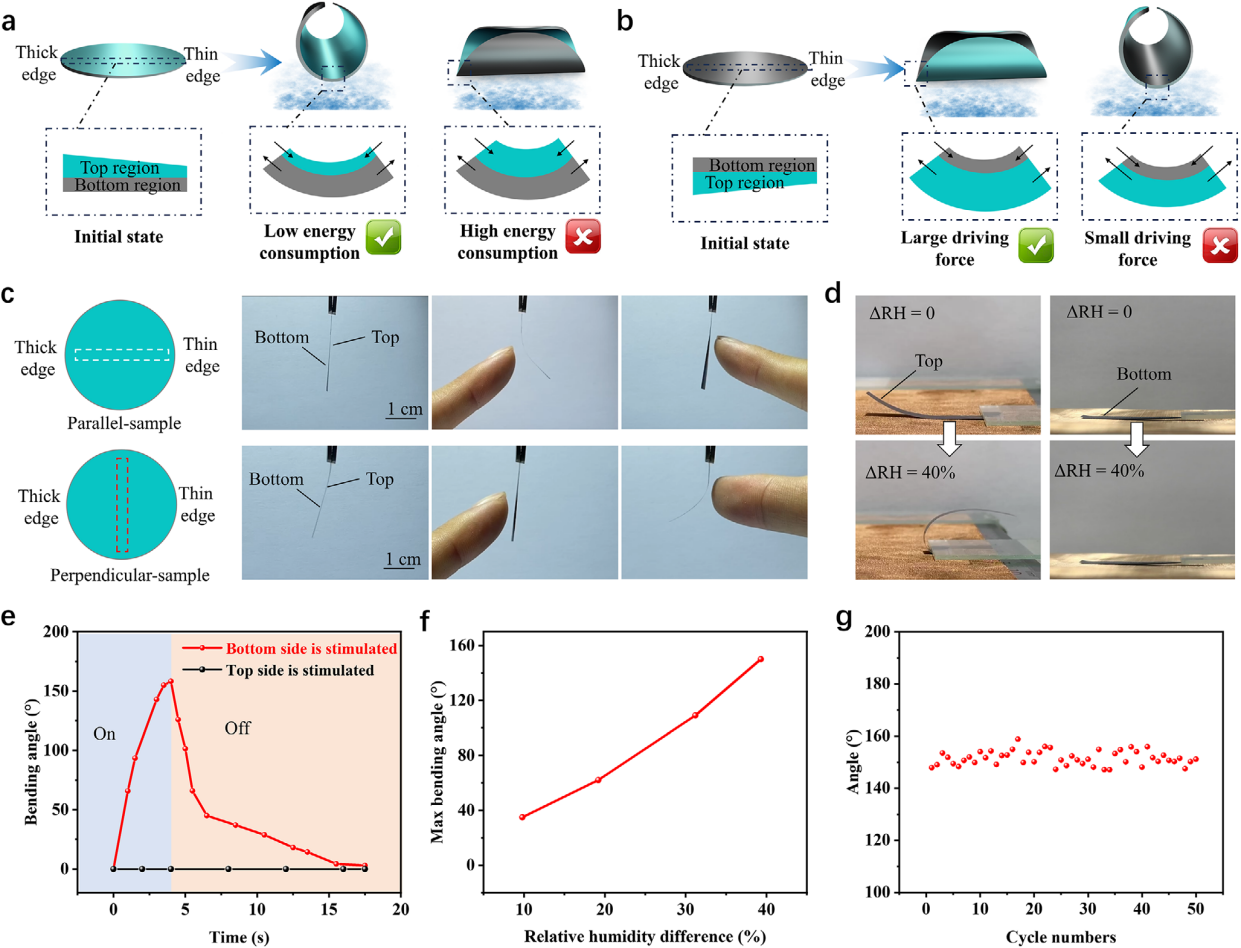
Deformation mechanism of the “diode‐like” actuation behavior and the actuation performance of the rectangular composite film samples. a) Actuation mechanism of the “diode‐like” actuation behavior of the MXene/CMCS composite film when the bottom side is stimulated and b) when the top side is stimulated. c) Deformation behavior of the parallel‐sample and perpendicular‐sample when a finger approaches. d) Deformation behavior of the parallel‐sample when its bottom and top sides are stimulated by moisture, respectively. e) Real‐time bending angle changes of the parallel‐sample under moisture stimulation. The relative humidity difference on both sides of the sample is 40%. f) Maximum bending angle of the parallel‐sample under different relative humidity differences on both sides when stimulated on the bottom side. g) Deformation stability of the parallel‐sample. The relative humidity difference on both sides of the sample varies cyclically between 0 and 40%.

The deformation mechanism of the “diode‐like” directional actuation behavior of this MXene/CMCS composite film is further analyzed. For ease of explanation, the MXene/CMCS composite film can be considered as a “pseudo bilayer” structure consisting of the top region and the bottom region. The pressure gradient generated during the vacuum‐assisted filtration process results in a denser stacking of MXene nanosheets at the bottom region, while the stack of MXene nanosheets at the top region is relatively loose. Therefore, the thickness gradient of the composite film is considered to exist in the top region. As shown in **Figure** **3**a, when water molecules stimulate the bottom region of the composite film, the volume of the bottom region expands while the volume of the top region remains approximately unchanged. Asymmetric deformation in the bottom and top regions will result in bending deformation of the composite film. If the bending axis is the symmetry axis parallel to the thickness gradient, the composite film needs to consume a large amount of driving energy to overcome the deformation limitation of the thicker part in the top region, which is unfavorable for the realization of the bending deformation. Therefore, considering the lowest energy principle, when water molecules stimulate the bottom region of the composite MXene/CMCS composite film with thickness gradient, the bending axis of the composite film is perpendicular to its thickness gradient direction. When water molecules stimulate the top region of the composite film, the top region changes from a deformation suppression layer to an active driving layer. The thicker part of the top region can generate greater driving force, thereby promoting the realization of bending deformation. Therefore, the bending axis of the composite film is the symmetry axis parallel to the thickness gradient direction when the top region is stimulated (Figure 3b).

In order to further confirm the correlation between the “diode‐like” actuation behavior of this MXene/CMCS composite film and its direction of thickness gradient and moisture source, the regions near the symmetry axis of perpendicular to and parallel to the thickness gradient direction of the composite film are cut to obtain rectangular samples (5 mm × 30 mm, select composite films from the same batch and cut them separately), and their moisture responsive actuation behavior are studied. The rectangular samples with a length direction parallel to the thickness gradient direction of the composite film are named “parallel‐samples”, and rectangular samples with a length direction perpendicular to the thickness gradient direction of the composite film are named “perpendicular‐samples”. As shown in Figure 3c, for the parallel‐sample, when a finger approaches the bottom side, the sample undergoes bending deformation away from the finger; when the finger approaches the top side, the sample does not show significant bending deformation. For the perpendicular‐sample, the situation is exactly the opposite: the significant bending deformation occurs when the finger approaches the top side of the sample. These results further demonstrate that the moisture responsive deformation behavior of this MXene/CMCS composite film is highly correlated with the direction of its thickness gradient and the direction of the moisture source.

The actuation behavior of rectangular samples with relative humidity differences at the bottom and top sides are further investigated. As shown in Figure 3d, a parallel‐sample is placed on a copper mesh with continuous moisture supply below, and the relative humidity difference between the lower area of the mesh and the upper air area is 40%. When the bottom side of the parallel‐sample contacts with the copper mesh, bending deformation can be achieved; while the top side of the sample contacts with the copper mesh, bending deformation does not occur. Figure 3e gives the real‐time bending angle changes of the parallel‐sample under moisture stimulation. When its bottom side is stimulated, the parallel‐sample undergoes bending deformation, with a bending angle exceeding 140° within 3 s. The maximum bending angles of the parallel‐sample under different relative humidity differences on both sides when stimulated on the bottom side are given in Figure 3f (the optical images are given in Figure S4, Supporting Information). With the increase of the relative humidity differences between the two sides of the sample, the bending angle of the sample increases. In addition, the moisture responsive actuation behavior of this MXene/CMCS sample exhibits good cyclic stability (Figure 3g). The actuation performances of the perpendicular‐sample are given in Figure S5 (Supporting Information), which is roughly similar to that of the parallel‐sample. The difference is that the bending deformation is occurred when its top side is in contact with the copper mesh.

### 2.3 Multifunctional Applications

**Figure 4:**
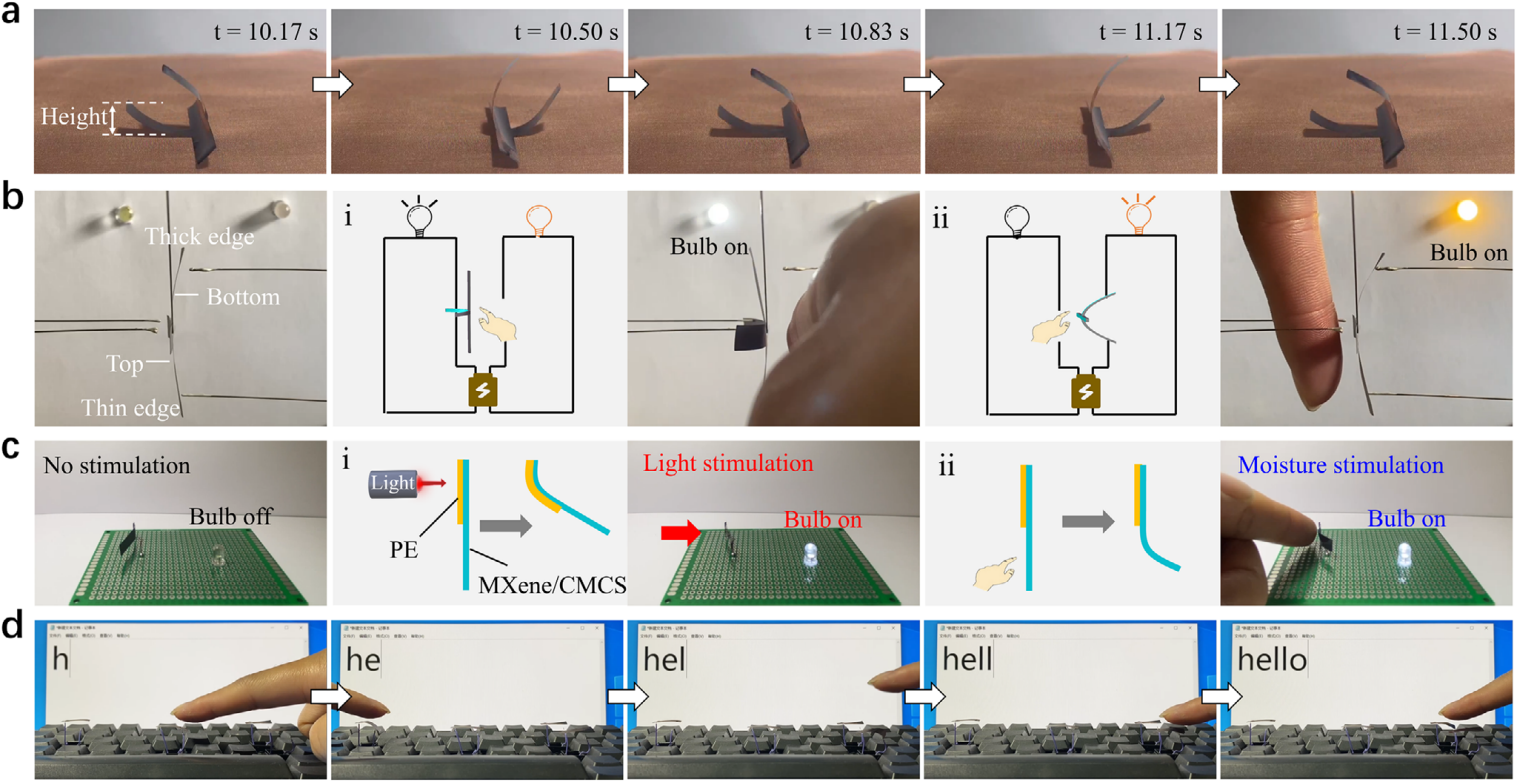
Multifunctional applications of the MXene/CMCS composite film actuator. a) Self‐sustained oscillation behavior of the cross‐shaped composite film sample under moisture gradient. b) Non‐contact switch with unidirectional conductivity. c) Dual‐responsive non‐contact switch. d) Intelligent keyboard for non‐contact character input.

Based on the excellent moisture responsive actuation performance of this MXene/CMCS composite film actuator, a series of intelligent multifunctional applications are demonstrated. The composite film is cut into a cross shape along the symmetry axis of perpendicular to and parallel to its thickness gradient direction and placed on a copper mesh with continuous moisture supply below. Thanks to the “diode‐like” actuation behavior of the composite film with gradient thickness, regardless of whether the top or bottom side of the cross shaped film is in contact with the copper mesh, the two arms on a same symmetry axis undergo upward bending deformation, while the other two vertical arms maintain their shape. Due to the unperfect symmetrical deformation of the two deformation arms, the center of gravity of the sample shifts and the sample tilts toward one side. The tilt of the sample causes the deformation arm on the tilted side to approach the moisture source, resulting in a larger driving deformation than the arm on the other side. This causes the center of gravity of the sample to shift again, and the sample tilts toward the other side. Subsequently, the deformation arm that originally had a large deformation amount moves away from the moisture source, and its deformation gradually decreases. The other deformation arm is approach to the moisture source, causing an increase in deformation. Then, the center of gravity of the sample shifts again, and the sample tilts to the opposite direction. The above processes occurred repeatedly, resulting in the cross shaped sample exhibiting self‐sustained oscillation behavior (**Figure** **4**a; Movie S2, Supporting Information). Figure S6 (Supporting Information) gives the height variation of the left deformation arm of the cross shaped sample during its self‐sustaining oscillation, which exhibits periodic fluctuations. More than 150 cycles of oscillation can be achieved within 2 min, and the average oscillation frequency of the sample exceeds 1 Hz. As shown in Figure S7 (Supporting Information), even after 15 min of continuous self‐oscillation (more than 1000 cycles), the oscillation performance of the sample remains stable.

In addition to achieving self‐sustained oscillation behavior, this cross shaped MXene/CMCS composite film actuator can also be used for a non‐contact switch with unidirectional conductivity similar to diodes (Movie S3, Supporting Information). In its initial state, the cross shaped film is not in contact with the metal conducts on both sides, and the circuit is in a disconnected state. When a figure approaches the right side (bottom side) of the cross shaped film, the two arms perpendicular to the thickness gradient direction of the film bend to the left and touch the two electrodes on the left side after ≈6 s. Due to the good conductivity (Figure S8, Supporting Information) of the MXene/CMCS composite film, the circuit where the white bulb is located is conducted, and the white bulb lights up (Figure 4b‐i). When a figure approaches the left side (top side) of the cross shaped film, the two arms parallel to the thickness gradient direction of the film bend to the right and touch the electrodes on the right side after ≈5 s. Then, the circuit where the orange bulb is located is conducted, and the orange bulb lights up (Figure 4b‐ii).

Combining this MXene/CMCS composite film with a polymer film, a multi‐responsive non‐contact switch can also be achieved. The half bottom side of a parallel‐sample is connected to a polyethylene film with adhesive to form a bilayer structure. Due to the high transmittance of polyethylene, the excellent photothermal conversion efficiency of the MXene/CMCS composite film, and the large difference in coefficient of thermal expansion between polyethylene film and MXene/CMCS composite film, when a laser beam is irradiated to the bilayer area from the left, the bilayer composite film bends toward the right side, which causes the right side of MXene/CMCS composite film to contact the electrode, the circuit is conducted, and the bulb lights up (Figure 4c‐i; Movie S4, Supporting Information). When the laser beam is removed, the bulb goes out. In addition to being able to control the switch with light stimulation, this non‐contact switch can also be controlled by moisture gradient. When a figure approaches to the MXene/CMCS composite film area from the left, this part can also bend toward the right side and the bulb can light up (Figure 4c‐ii). This MXene/CMCS composite film driven by moisture is also used to develop an intelligent non‐contact keyboard. Lead out the electrode wires on the upper and lower plates of the keyboard, and connect the electrode wires on the upper plate to the MXene/CMCS composite film through silver conductive adhesive. When a finger approaches the composite film, it bends and connects the circuit of the upper and lower plates, and continuous character editing on the computer without physical contact can be achieved (Figure 4d; Figure S9 and Movie S5, Supporting Information). Based on this MXene/CMCS composite film, a biomimetic inchworm crawling robot is also designed and fabricated. By repeatedly approaching/moving away from the robot with human finger, the robot can achieve crawling motion on a serrated platform (Figure S10 and Movie S6, Supporting Information). In addition, a smart wearable clothing with sweat‐responsive switch composed of MXene/CMCS composite films is designed for personal dynamic thermal management, which can adaptively promote sweat evaporation and eliminate body temperature rise (Figure S11, Supporting Information). When the human body feels comfortable in the environment, there is no excess sweat evaporating from the body surface, and the sweat‐responsive switch is in a closed state. When the human body sweats due to high environmental temperatures or during exercise, a large amount of sweat is produced on the body surface, and the sweat‐responsive switch can automatically open to evaporate sweat and promote convection.

**Figure 5:**
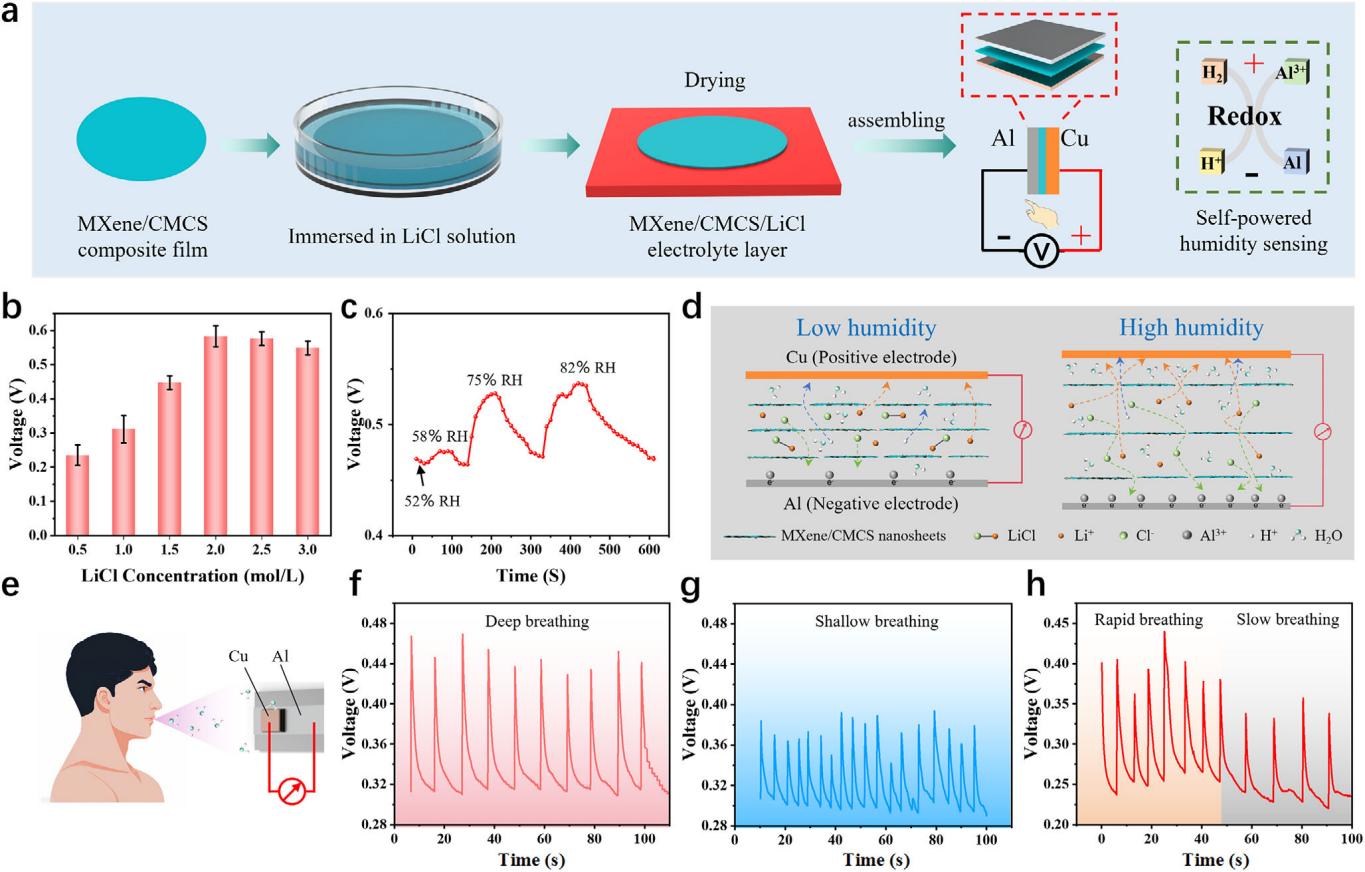
Application of the MXene/CMCS composite film in self‐powered moisture‐electric humidity sensor. a) Schematic illustration of the fabrication procedure of the self‐powered moisture‐electric humidity sensor. b) The influence of the concentration of lithium chloride solution on the output voltage of the obtained sensor. c) The output voltage of sensors obtained after treatment with LiCl solutions of different concentrations at 99% RH. d) Humidity sensing mechanism of the sensor. e) Schematic diagram of the sensor used for respiratory monitoring. f) The output voltage of the sensor under deep breathing and g) shallow breathing conditions. h) Real‐time output voltage of the sensor under rapid and slow breathing conditions.

Based on this MXene/CMCS composite film, a self‐powered moisture‐electric humidity sensor for monitoring changes in human respiration is also designed and fabricated. **Figure** **5**a gives the schematical diagram of the fabrication process of the sensor. Simply put, the MXene/CMCS composite film is immersed in lithium chloride (LiCl) solution for a period of time, and then dried in an oven to obtain the MXene/CMCS/LiCl electrolyte layer. After that, the MXene/CMCS/LiCl electrolyte layer is sandwiched between an aluminum (Al) foil electrode and a copper (Cu) foil electrode to construct the self‐powered moisture‐electric humidity sensor. This sensor exhibits similar characteristics to primary batteries in terms of functional structure and chemical reactions. When the sensor is in a humid environment, the MXene/CMCS/LiCl electrolyte layer can adsorb water molecules from the air. The adsorbed water molecules undergo ionization, producing a large amount of H^+^ and OH^‐^ as charge carriers, and also promoting the dissociation of LiCl, producing Li^+^ and Cl^‐^. The active Al negative electrode loses electrons and converts to Al^3+^. The H^+^ in the electrolyte receives electrons at the Cu positive electrode and transforms into H_2_, thereby generating an electric field. Then, Li^+^ and Cl^‐^ in the MXene/CMCS/LiCl electrolyte directionally migrate to the positive and negative electrodes in the electric field, respectively, forming a conductive path and simultaneously generating current in the external circuit. This redox reaction is the energy source for the self‐powered humidity sensor. Movie S7 (Supporting Information) shows that a significant and rapid increase in output voltage can be observed when approaching the device with a figure, indicating the potential application of the device as a humidity sensor. The effect of LiCl concentration on the output voltage of the sensor is studied to obtain the optimal fabrication process. As shown in Figure 5b, when the LiCl concentration is below 2 mol L^−1^, the output voltage of the sensor increases with the increase of LiCl concentration and reaches its maximum value (≈5.8 V, 99% RH) at 2 mol L^−1^. When the concentration exceeds 2 mol L^−1^, the output voltage of the sensor slightly decreases. The results can be attributed to the highly concentrated LiCl particles occupying the ion migration space, hindering ion migration, and thus reducing the output voltage. Therefore, a 2 mol/L LiCl solution is chosen to fabricate the self‐powered humidity sensor.

Thanks to the excellent hydrophilicity of MXene and CMCS, as well as the good hygroscopicity and ionization properties of the ion provider LiCl, the redox reaction in this primary battery structure is highly sensitive to humidity. The humidity changes can be monitored by measuring the open circuit voltage of the sensor without an external power source. The dynamic response and recovery process of the output voltage of the sensor under different humidity environments are given in Figure 5c. It can be seen that with the increase of environmental humidity, the output voltage of the sensor gradually increases. The humidity sensing mechanism of this sensor is shown in Figure 5d. Under low humidity conditions, it is difficult to form a continuous ion conductive path between the electrodes, which hinders the movements of Li^+^ and Cl^‐^ in the electrolyte. Therefore, the electrode potential difference is small, and the output voltage of the sensor is low. Under high humidity conditions, due to the excellent hygroscopicity of MXene and CMCS, the electrolyte layer absorbs more water molecules and expands in volume, reducing the steric hindrance of ion transport. In addition, the abundant water molecules enhance the hydration and mobility of ions. Therefore, an increase in humidity can intensify redox reactions, increase ion mobility, and thus generate higher voltage output. Figure S12 (Supporting Information) gives the stability of the power generation performance of this humidity sensor at 60% RH. Within 10 000 s, the output voltage of the sensor remains around 0.5 V, indicating its stable and sustainable power generation capacity. Finally, the application of this humidity sensor in respiratory monitoring is demonstrated (Figure 5e). As shown in Figure 5f, the gas produced by deep breathing has a higher relative humidity, therefore, the sensor exhibits a higher output voltage. In contrast, shallow breathing shows a relatively lower output voltage (Figure 5g). The real‐time voltage signals of rapid and slow breathing are also recorded, indicating that this humidity sensor also has the ability to recognize the speed of breathing (Figure 5h). The above‐mentioned actuating applications based on finger metabolism of water and sensing application based on human respiration were achieved under unified laboratory conditions (≈25 °C, 50%≈55% RH). Considering that the MXene‐based materials can be saturated with moisture under high humidity conditions, the applicable environmental humidity range for the relevant applications has been further confirmed. When the environmental relative humidity is within 70%, the above applications can be successfully reproduced.

## 3 Conclusion

In summary, we develop a simple, universal, and low‐cost vacuum‐assisted “tilt‐filtration” method to fabricate a Ti_3_C_2_T_x_ MXene/carboxymethyl chitosan composite film actuator with thickness gradient. The introduction of carboxymethyl chitosan improves the mechanical properties of MXene and increases its interlayer spacing and water absorption expansion capability. Thanks to the high sensitivity of MXene/carboxymethyl chitosan composite material to moisture changes, this composite film actuator can generate rapid driving deformation under moisture gradient environment. More importantly, the asymmetric internal structure causes the actuator to exhibit a “diode‐like” controllable unidirectional deformation behavior under moisture gradient, and its deformation direction is strictly correlated to its thickness gradient direction and the moisture source direction. Based on its “diode‐like” controllable unidirectional deformation behavior, a moisture responsive self‐oscillator based on this composite film with thickness gradient is achieved. Under a constant moisture gradient environment, the oscillator can realize self‐sustained oscillating motion. In addition, based on the excellent moisture response of this composite film actuator, it can also be used in various intelligent devices driven by human metabolism, such as non‐contact switches with unidirectional conductivity and light/moisture dual response, intelligent keyboard for non‐contact character input, soft biomimetic crawling robot, wearable intelligent thermal management clothing, and self‐powered respiratory sensor. This work provides a simple and novel universal strategy for designing monolithic actuators with controllable unidirectional deformation, and is expected to promote the further development of sustainable, green, and low‐energy moisture responsive soft actuators in soft robotics and electronics.

## 4 Experimental Section

### Synthesis of Ti_3_C_2_T_x_ MXene Suspension

The T_3_C_2_T_x_ MXene was synthesized using the HCl/LiF chemical etching method. Specifically, 3.2 g LiF was dissolved into 30 mL 9 mol L^−1^ HCl, and then 2 g Ti_3_AlC_2_ was slowly added to the mixed solution while continuously stirring the mixture. After reacting at 35 °C for 24 h, centrifuge the mixture at 3500 rpm for 5 min to obtain the etching product. Then, etching product was washed the multiple times with deionized water until the pH value of the upper liquid was >6. After that, centrifuge the aqueous dispersion of the product at 3500 rpm for 30 min, and the upper liquid was collected to obtain the T_3_C_2_T_x_ MXene suspension.

### Fabrication of the MXene/CMCS Composite Film Actuator

In this work, the optimal mass ratio of MXene to CMCS is 10:1, at which the composite film exhibits the best mechanical properties and actuation performance. 3 mg CMCS was dissolved in 100 mL deionized water and sonicated for 30 min to obtain the CMCS aqueous solution. Subsequently, slowly added 6 mL MXene dispersion (5 mg mL^−1^) to the CMCS aqueous solution and continue magnetic stirring for 30 min. After that, the mixed solution was poured into the filtration device and perform vacuum‐assisted tilt‐filtration to obtain the MXene/CMCS composite film. Finally, the obtained composite film should be naturally dried and removed from the filter membrane for later use.

### Characterization

The TEM image of MXene nanosheet was obtained by a field emission transmission electron microscope (JEM‐2100F, JEOL, Japan). The SEM images of the samples were obtained by a field emission scanning electron microscope (SU8000, Hitachi, Japan). The XRD patterns of T_3_C_2_T_x_ MXene and MXene/CMCS were obtained by an X‐ray diffractometer with high‐intensity graphite monochromatized Cu Kα radiation (Rigaku‐TTR3, Japan). The FTIR spectra were measured by a Fourier transform IR spectrometer (Nicolet iN10, ThermoFisher, America). The mechanical properties of pure MXene film and MXene/CMCS composite film were tested by a universal mechanical testing machine (5944, Instron, America). The authors confirmed that informed written consent from human research participant was obtained prior to the research.

## Conflict of Interest

The authors declare no conflict of interest.

## Supporting information



Supporting Information

Supplemental Movie 1

Supplemental Movie 2

Supplemental Movie 3

Supplemental Movie 4

Supplemental Movie 5

Supplemental Movie 6

Supplemental Movie 7

## Data Availability

The data that support the findings of this study are available from the corresponding author upon reasonable request.
