# Supplementary material for: MXene/Carboxymethyl Chitosan Moisture Responsive Soft Actuator with Diode‐Like Actuation for Versatile Applications Driven by Human Metabolism
Source: Adv Sci (Weinh). 2025 Jul 21;13(15):e07845. doi: 10.1002/advs.202507845 (PMC13042369; doi:10.1002/advs.202507845)
Supplement: Supplementary file 1 — Supporting Information [file ADVS-13-e07845-s008.docx]

Supporting Information

**MXene/Carboxymethyl Chitosan Moisture Responsive Soft Actuator with Diode-Like Actuation for Versatile Applications Driven by Human Metabolism**

*Liangliang Xu, Yangyang Ling, Ziqing Li, Xinyu Xu, Xiaoxia Li, Longfei Chang*, Qingyu Peng*, and Ying Hu**

**Supplemental Figures**


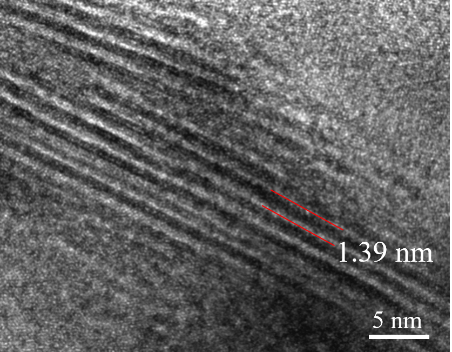


**Figure S1** HRTEM image of Ti_3_C_2_T_x_ MXene nanosheet.


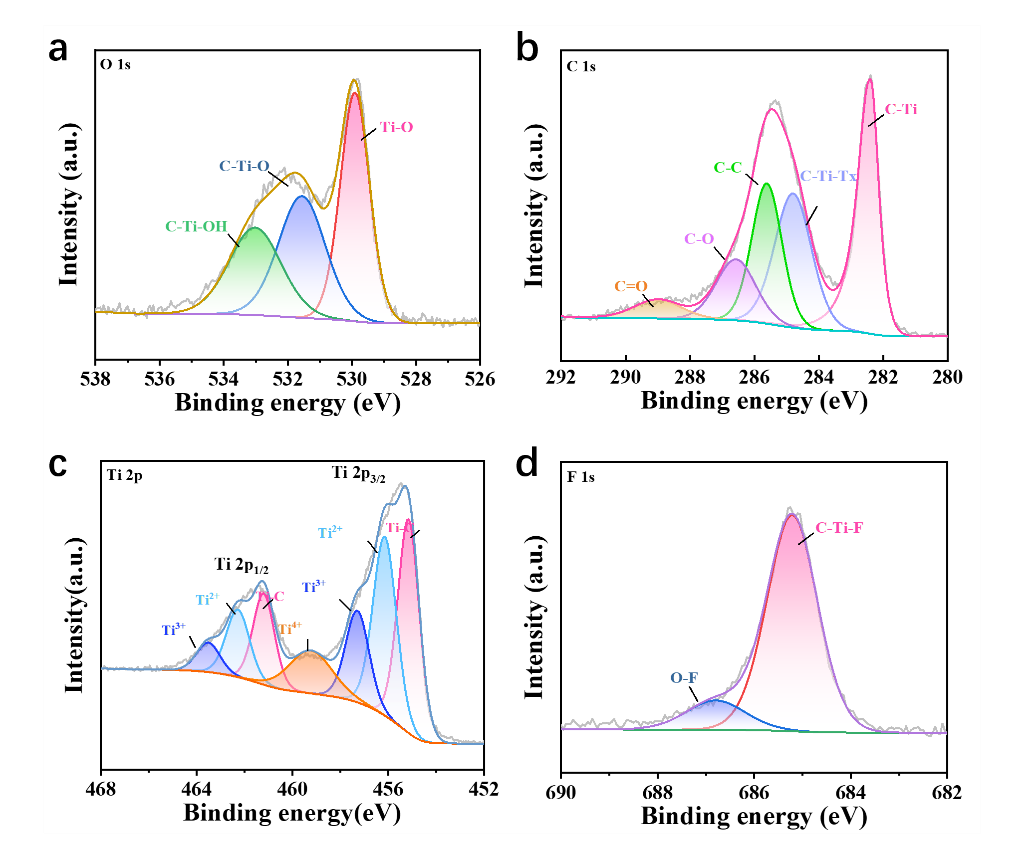


**Figure S2** High-resolution XPS spectra of Ti_3_C_2_T_x_ MXene in (a) O 1s, (b) C 1s, (c) Ti 2p, and (d) F 1s regions.


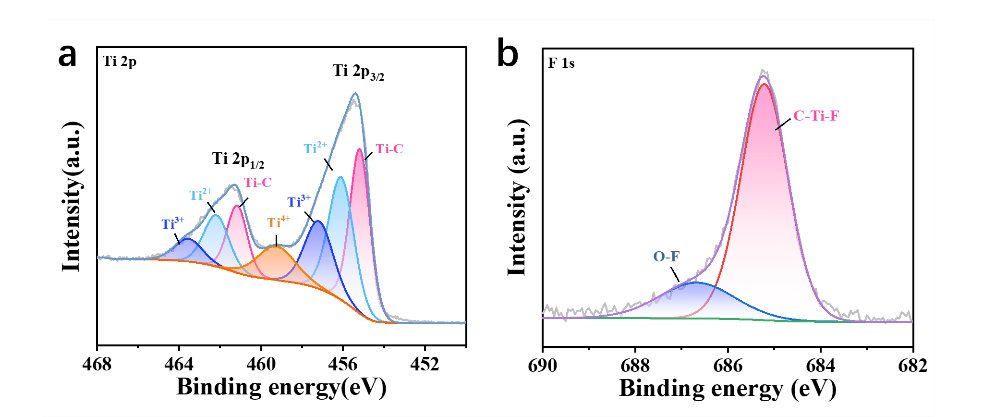


**Figure S3** High-resolution XPS spectra of MXene/CMCS composite material in (a) Ti 2p and (b) F 1s regions.


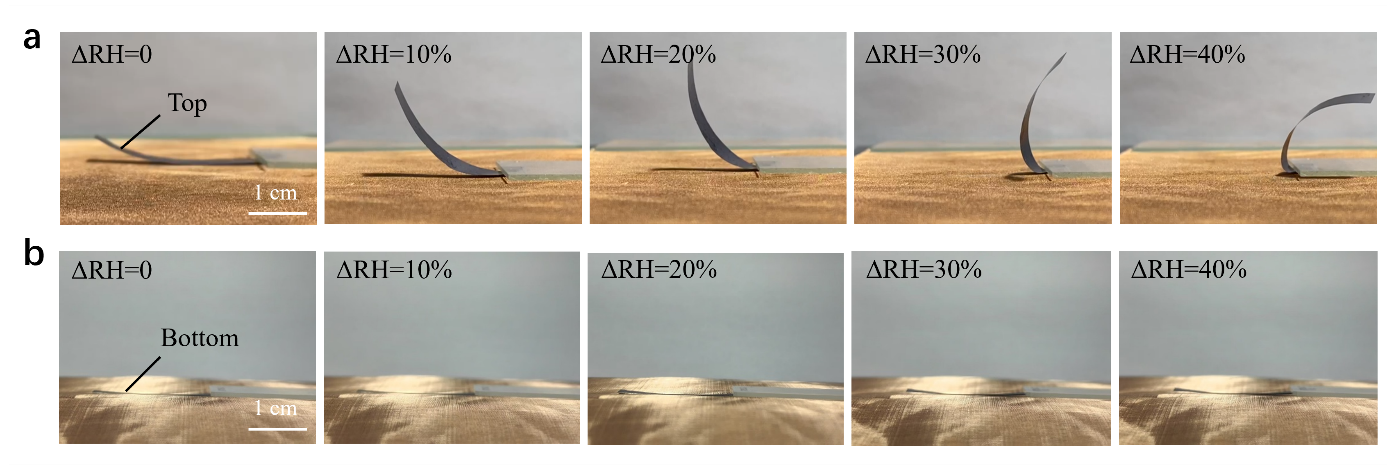


**Figure S4** Optical images of the parallel-sample under different relative humidity differences on both sides. (a) The bottom side is stimulated by moisture. (b) The top side is stimulated by moisture.


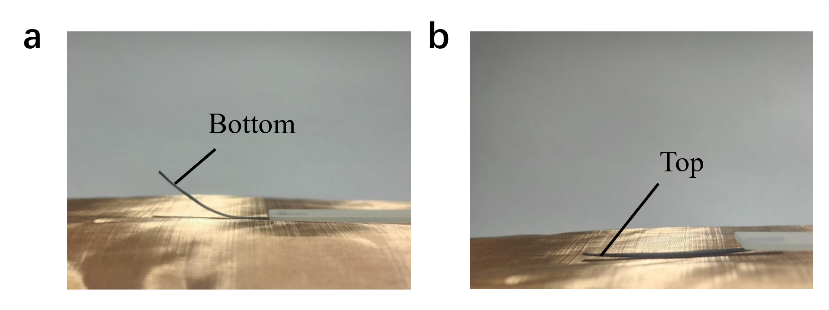


**Figure S5** Optical images of the perpendicular-sample under moisture stimulation. (a) The top side is stimulated. (b) The bottom side is stimulated.





**Figure S6** The height variation of the right top of the cross shaped sample during its self-sustained oscillation process.


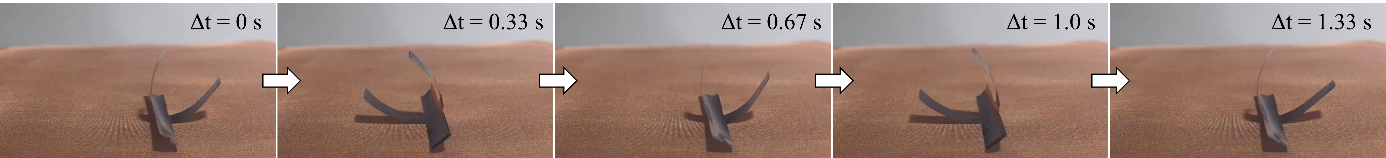


**Figure S7** Stable self-sustained oscillation process of the cross shaped composite film sample under moisture gradient after more than 15 min oscillation.


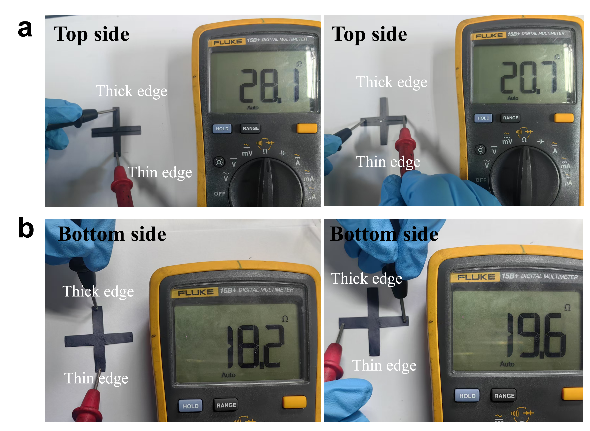


**Figure S8** Resistance tests of the cross shaped MXene/CMCS composite film. (a) Top side, (b) bottom side.


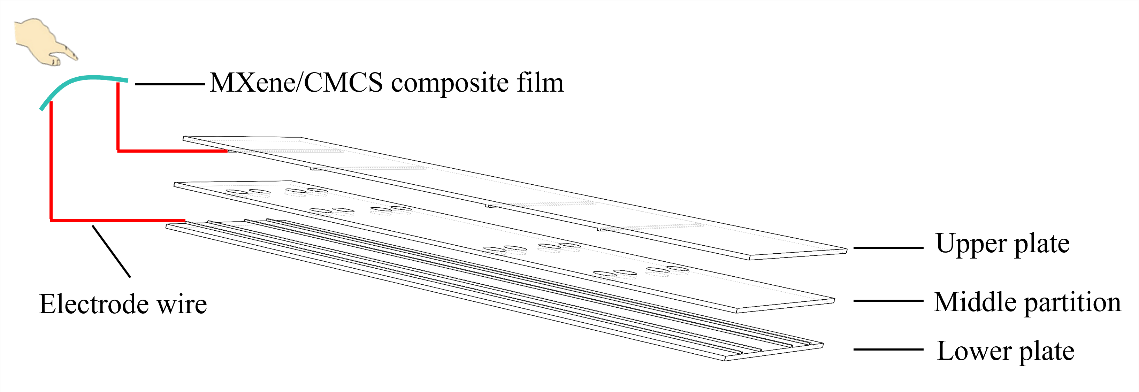


**Figure S9** Schematic diagram of the non-contact intelligent keyboard operation.


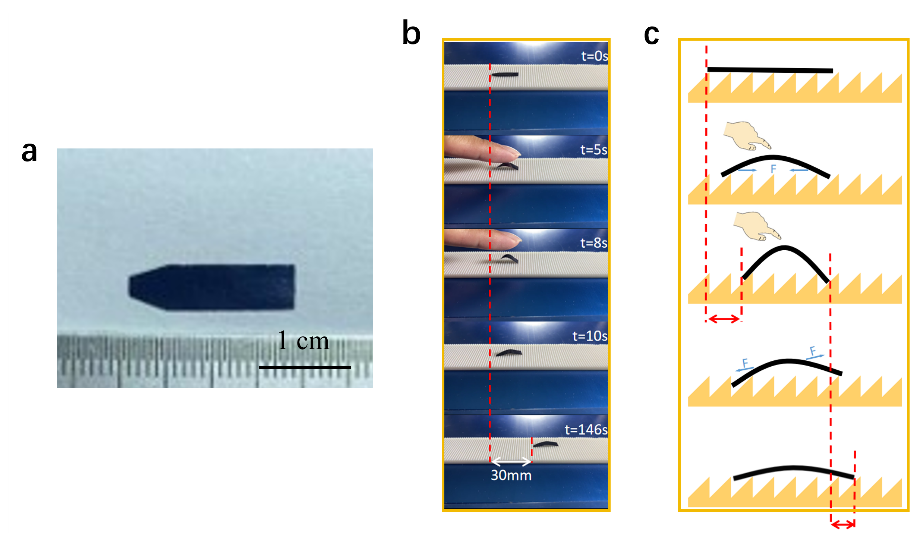


**Figure S10** (a) Optical image of the biomimetic crawling robot. (b) Optical images of the crawling motion process of the biomimetic robot. (c) Schematic diagram of the movement mechanism of the crawling robot.


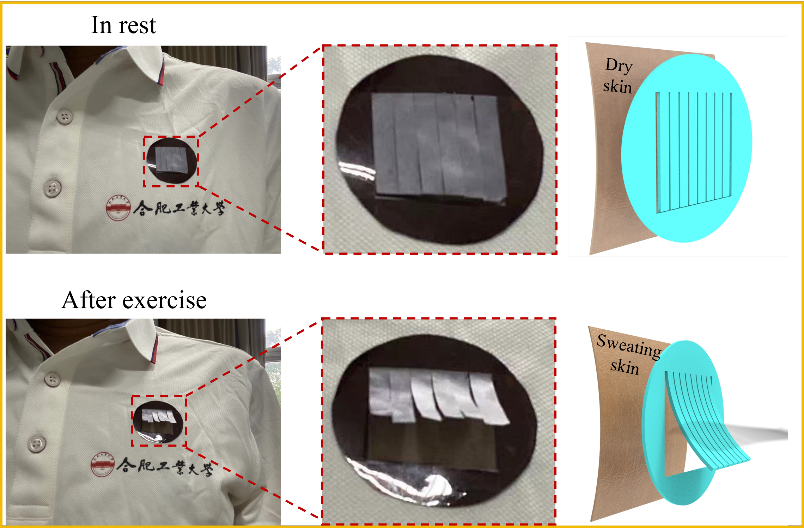


**Figure S11** Smart wearable clothing with sweat-responsive switch composed of MXene/CMCS composite films.


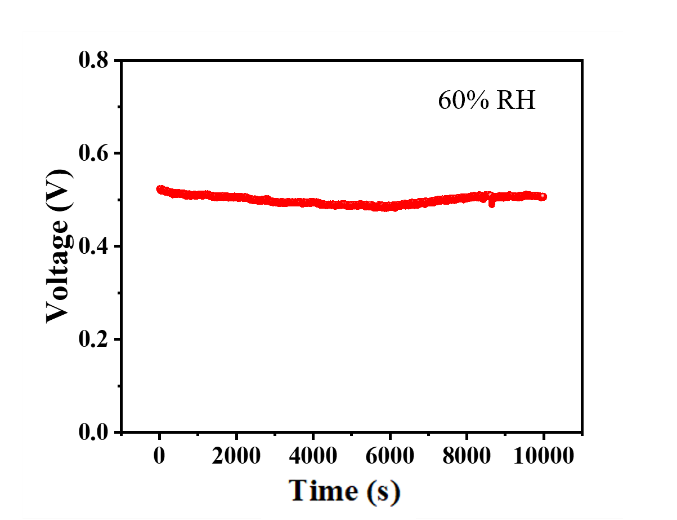


**Figure S12** Stability of the power generation performance of the sensor at 60% RH.

**Supplemental Videos**

**Movie S1** Periodic flipping motion of the composite film under continuous moisture stimulation.

**Movie S2** Self-sustained oscillation behavior of the cross shaped sample under continuous moisture stimulation.

**Movie S3** A non-contact switch with diode-like unidirectional conductivity.

**Movie S4** A moisture/light dual-responsive non-contact switch.

**Movie S5** An intelligent keyboard for non-contact character input.

**Movie S6** A biomimetic crawling robot.

**Movie S7** Real-time response of the self-powered humidity sensor when a human finger approaches.
